# Modified sensory feedback enhances the sense of agency during continuous body movements in virtual reality

**DOI:** 10.1038/s41598-021-82154-y

**Published:** 2021-01-28

**Authors:** Kei Aoyagi, Wen Wen, Qi An, Shunsuke Hamasaki, Hiroshi Yamakawa, Yusuke Tamura, Atsushi Yamashita, Hajime Asama

**Affiliations:** 1 Department of Precision Engineering, The University of Tokyo, 7-3-1 Hongo, Bunkyo-ku, Tokyo, 113-8656 Japan; 2 Faculty of Information Science and Electrical Engineering, Kyushu University, Fukuoka, Japan; 3 Department of Robotics, Tohoku University, Sendai, Japan

**Keywords:** Psychology, Engineering

## Abstract

The sense of agency refers to the feeling of control over one’s own actions, and through them, the external events. This study examined the effect of modified visual feedback on the sense of agency over one’s body movements using virtual reality in healthy individuals whose motor control was disturbed. Participants moved a virtual object using their right hand to trace a trajectory (Experiment 1) or a leading target (Experiment 2). Their motor control was disturbed by a delay in visual feedback (Experiment 1) or a 1-kg weight attached to their wrist (Experiment 2). In the offset conditions, the virtual object was presented at the median point between the desired position and the participants’ actual hand position. In both experiments, participants reported improved sense of agency in the offset condition compared to the aligned condition where the visual feedback reflected their actual body movements, despite their motion being less precise in the offset condition. The results show that sense of agency can be enhanced by modifying feedback to motor tasks according to the goal of the task, even when visual feedback is discrepant from the actual body movements. The present study sheds light on the possibility of artificially enhancing body agency to improve voluntary motor control.

## Introduction

When individuals navigate their physical environments, they experience a sense of control over their actions, and through that, over the external events. This subjective feeling of control is called the sense of agency or the sense of control^1^. Studies in cognitive science have found that the sense of agency influences many aspects of behavior, such as perception^2–4^, attention^5–7^, and decision making^8,9^. Research on the sense of agency has attracted the interest of researchers in many fields besides cognitive science, such as robotics^10–12^ and psychiatry^13–16^.

**Figure 1:**
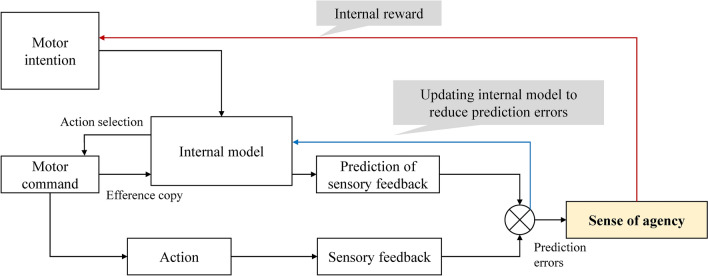
The model of voluntary motor control depicting the role of the sense of agency. According to the comparator model^3^, the sense of agency is generated from the comparison between predicted sensory feedback and actual sensory feedback. Prediction errors reduce the sense of agency, and cause the updating of the internal model for motor control. The sense of agency is also considered as an internal reward for voluntary motor control^18,19^.

The sense of agency is usually generated from the comparison between the predictions of sensory feedback based on the efference copy of one’s motor commands, and the actual sensory feedbacks^3^ (Fig. 1). Prediction errors (i.e., mismatch between predictions and sensory feedbacks) diminish the sense of agency and trigger the update of the internal model and action selection to reduce prediction errors^17^. Sense of agency is considered an awareness accompanying motor control. However, recent studies have shown that the sense of agency is more than a passive awareness. Feeling in control reduces the reaction time of executing an action, and increases the frequency of action^18,19^. Furthermore, a recent study showed that when people controlled a virtual limb and felt that the virtual limb was a part of their own body, the movements of their real limb was “attracted” to the movements of the virtual limb^20^. In other words, feeling in control is not only a passive “side effect” of control, but can affect motor control itself.

This study focuses on the sense of agency during continuous body movement when one’s motor control is disturbed. When we move our hands, we usually undoubtedly know that we are controlling our hands without any conscious effort. However, the sense of agency over one’s body may be impaired when one’s motor ability is impaired. For example, after stroke, many patients not only suffer from motor disabilities such as apraxia and hemiparetic/-plegic limbs, but also report abnormal self-awareness of action^16,21,22^. This is probably because patients’ internal model for controlling their own body remains at the old state before the impairment, and large prediction errors arise when they try to execute an action. Many rehabilitation studies have been conducted to improve the voluntary skillful movement of hemiplegic patients^23^, using physical techniques such as exercises, massage, and manipulation. This can physically reduce prediction errors by improving the control of one’s limbs. However, because of the great difficulty of motor recovery, this progress is usually slow and frustrating. Here, this study sheds light on the possibility of intervening sensory feedback. We suggest that positive intervention in sensory feedback can artificially reduce prediction errors and improve the sense of agency, potentially enhancing the motivation of action. Furthermore, reducing large prediction errors may also be beneficial for the updating of the internal model to adapt to the current state of body motor control after impairment^24,25^. Some previous studies attempted to use extrinsic feedback to improve motor learning in stroke patients by providing supplementary sensory information to reduce the gap between the expected state of motion and the actual motion^26–28^. For example, visual feedback about weight distribution can improve patients’ balance performance, and auditory feedback of force production can improve sit-to-stand performance^26^. Here, this study examined whether a modification of sensory feedback that reduces perceived prediction errors could enhance the sense of agency, even when the actual motor errors are still large. If this hypothesis is valid, it means that external modification of sensory feedback may be a useful way to enhance body agency, and potentially benefit voluntary motor learning for people with motor disabilities.

In this study, we designed two experiments with a motor task with virtual reality (VR), using a head-mounted display (HMD), which allowed us to prevent the participants from seeing their own body, and therefore to replace the visual input of the body with modified visual feedback. Participants moved their hand to trace a designated trajectory (Experiment 1) or a leading stimulus (Experiment 2). The position of their hand was presented by a visual stimulus (i.e., a ball), which participants believed and felt matched the position of their real hand in real space. Although the visual stimulus was an external object instead of one’s real body, controlling this object relies on exactly the same internal model as controlling one’s own hand, requiring no spatial or temporal transformation, thus the sense of agency over the visual stimulus in our task is very close to the sense of agency over one’s own body movements. Participants’ motor control was disturbed by a delay in visual feedback (Experiment 1) or a 1-kg weight attached to their wrist (Experiment 2). In our offset conditions, the visual feedback of the hand position was presented at the median point between participants’ actual hand position and the desired position (according to the goal of each experiment, see method of each experiment). We predict that the matches between participants’ motor intention and visual feedback should improve the sense of agency, even when the actual motion errors are large.

## Experiment 1

### Participants

Fifteen participants took part in Experiment 1 (4 females; mean age = 21.6 years; standard deviation, *SD* = 2.4). All participants had normal or corrected-to-normal visual acuity and normal motor ability, and were right-handed. The experiment was conducted with the approval of the ethics committee of the Faculty of Engineering at the University of Tokyo, and was performed in accordance with relevant guidelines and regulations. Written informed consent was obtained from all participants.

### Experimental task and design

**Figure 2:**
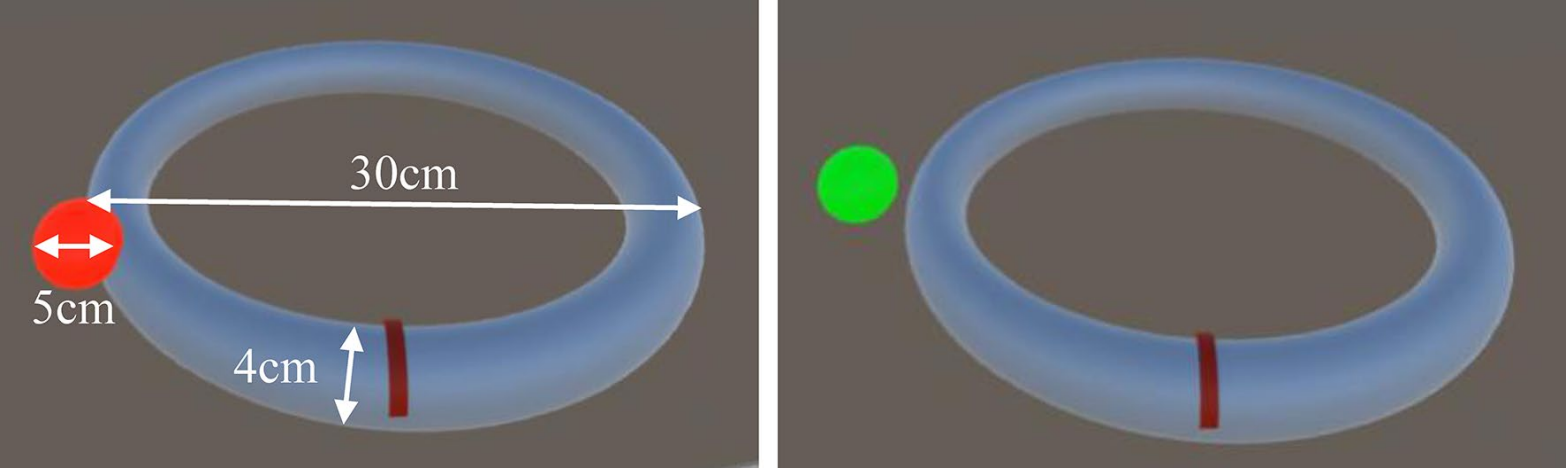
The trajectory and visual stimulus (i.e., the ball) used in Experiment 1. The visual stimulus represents the position of the participant’s hand. The color of the visual stimulus was green when it was not touching the trajectory (right panel), and was red when it was touching the trajectory (left panel).

In the experiment, a circular trajectory and a spherical stimulus were presented in the virtual space with the HMD (Fig. 2). The circular trajectory was presented on a horizontal surface in the virtual space in front of the participants. The diameter of the trajectory and the diameter of the visual stimulus were 30 cm and 5 cm, respectively. The position of the visual stimulus (i.e., the ball in Fig. 2) was synchronized with the position of the participants’ right hand using a motion capture. The color of the visual stimulus was green when it was away from the trajectory and changed to red when it touched the trajectory. The participants were instructed to move their right hand on this trajectory, maintaining the visual target as close to the trajectory as possible (i.e., keeping the ball red). The participants were instructed to move their hand at a speed of 4 s per circle. A metronome of 4 s was played during the task. The participants moved four circles in each trial. After each trial, they rated, on a 7-point scale from 1 (not at all) to 7 (very much), for the question “How much did you feel that the ball in VR was controlled by your hand movements?”.

**Figure 3:**
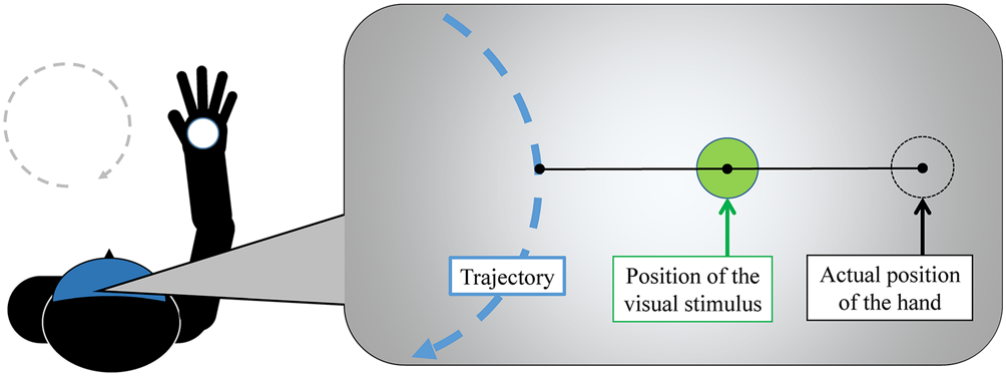
The position of the visual stimulus in the *offset* condition.

There were two within-individual factors: delay in visual feedback and visual modification. In the *delayed* condition, there was a time lag of 600 ms in the visual feedback of participants’ hand positions. This time lag was selected according to our pilot experiments, confirming that a delay of 600 ms was sufficient to reduce the sense of agency in the experimental task. In the *no-delay* condition, the position of the visual stimulus represented the position of the participant’s hand in real-time. For visual modification, the position of the visual stimulus was either aligned or offset from the hand position. In the *aligned* condition, the visual stimulus was presented at the actual hand position. In the *offset* condition, the visual stimulus was presented at the median point between the actual hand position and the closest point on the trajectory to the hand position (Fig. 3). These two factors in a 2 × 2 design resulted in four conditions: no-delay and aligned, no-delay and offset, delayed and aligned, and delayed and offset. We predicted that delay would produce more motion errors and reduce the sense of agency, while modified visual feedback would improve the sense of agency, especially in the delayed condition.

### Devices and procedure

**Figure 4:**
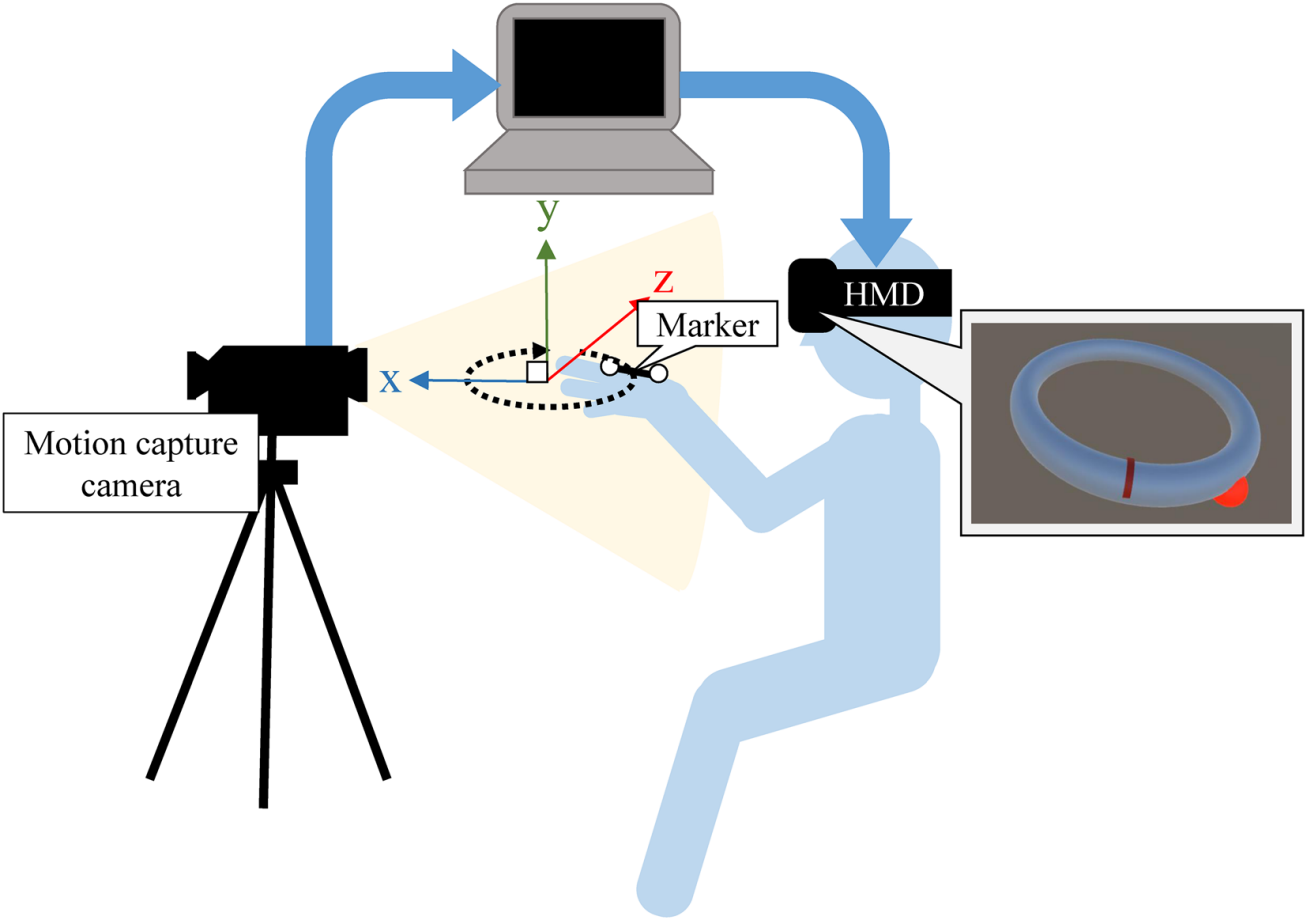
Configuration of the system in Experiment 1.

Figure 4 shows the configuration of the system. The experimental system contained an HMD (Oculus Rift, Facebook Technologies, LLC), a motion capture (OptiTrack V120: Trio, NaturalPoint, Inc.), and a desktop computer. A marker for motion capture was attached to the back of the participants’ right hand during the experiment. Unity (2017.2.0f3., Unity Technologies) was used to program the task and present the stimuli.

The experiment was conducted individually in a quiet room. Participants were first introduced to the devices. After wearing the HMD and getting the marker attached to their hands, the participants had two practice trials without any delay in visual feedback. They were told to move the visual stimulus by moving their hand to trace the trajectory as precisely as possible (i.e., maintaining the color of the visual stimulus red as much as possible). They were also told to listen to the metronome, and to try to move their hand at a speed of 4 s per circle. Each condition was repeated three times, resulting in 12 trials in total for each participant. The trial order was randomized. The experiment took 60 min on average for each participant.

### Results

#### Agency rating

**Figure 5:**
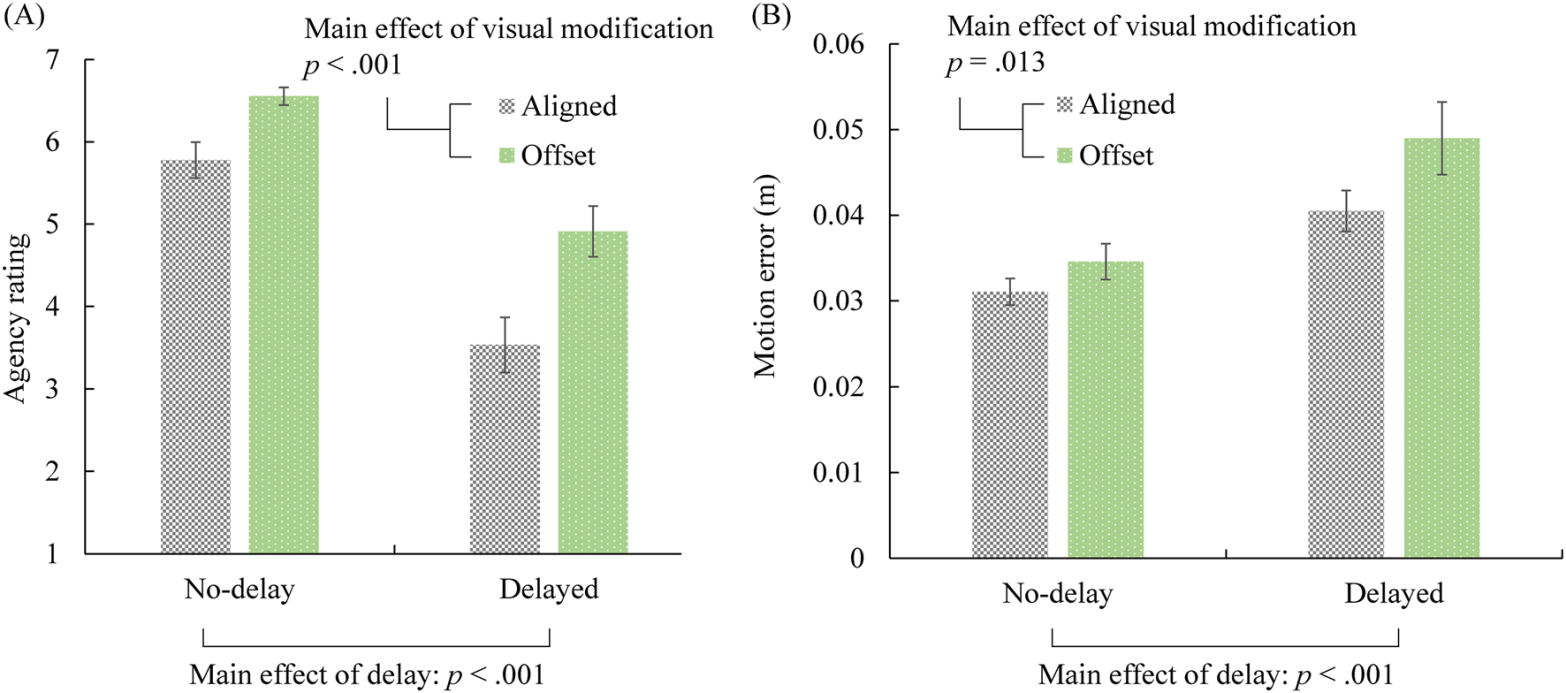
Agency rating (**A**) and motion error (**B**) in each condition in Experiment 1. Error bars represent standard errors. Delay in visual feedback decreased agency rating and increased motion error. On the other hand, modification of visual feedback increased agency rating as well as motion error.

Figure 5A shows the agency rating for each condition. The normality of agency rating in all four conditions was confirmed using Kolmogorov–Smirnov test. A 2 × 2 (delay × visual modification) repeated measures ANOVA on agency rating revealed a significant main effect of delay (*F*(1, 14) = 46.003, *p* < 0.001, partial η^2^ = 0.767), and a significant main effect of visual modification (*F*(1, 14) = 36.631, *p* < 0.001, partial η^2^ = 0.723). The interaction between delay and visual modification did not reach significance (*F*(1, 14) = 3.574, *p* = 0.080, partial η^2^ = 0.203). The results showed that delay greatly reduced the sense of agency, as predicted. More importantly, visual modification significantly improved the sense of agency. The results supported our main hypothesis that visual modification, which reduced visual prediction errors (see the section on visual prediction errors), enhances the sense of agency, even when the visual feedback does not reflect the actual body movements.

#### Motion error

**Figure 6:**
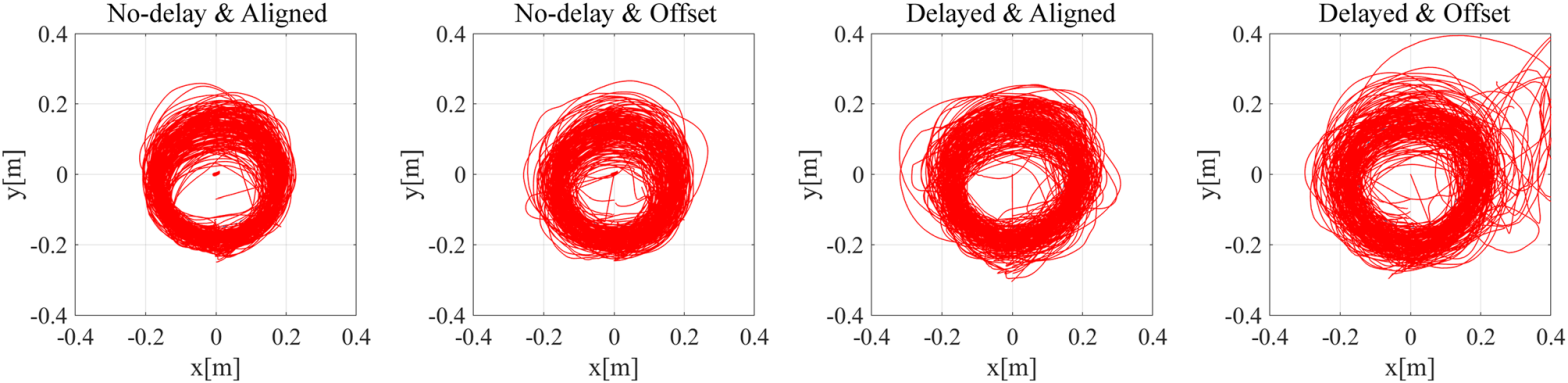
The participants’ moving trajectories plotted on the horizontal surface. The motion was more precise when there was no delay and when the visual feedback was not modified.

Visual modification was designed to reduce the visual prediction error. However, because the visual stimulus did not actually show the exact position of the hand in the offset condition, the participants’ motion could be less precise than in the aligned condition. Figure 5B shows the motion error in each condition, and Fig. 6 shows the actual trajectory of the hand movement in each condition. The motion error was the average distance between the actual hand position and the trajectory. The smaller the motion error, the more precisely the participants’ hand traced the trajectory. The normality of motor error in all four conditions was confirmed using Kolmogorov–Smirnov test. A 2 × 2 (delay × visual modification) repeated measures ANOVA on motion error revealed a significant main effect of delay (*F*(1, 14) = 22.009, *p* < 0.001, partial η^2^ = 0.611) and a significant main effect of visual modification (*F*(1, 14) = 8.190, *p* = 0.013, partial η^2^ = 0.369). The interaction between delay and visual modification was not significant (*F*(1, 14) = 2.991, *p* = 0.106, partial η^2^ = 0.176). The results showed that the error was indeed larger when the visual feedback was modified and when the delay in response was longer.

#### Visual prediction error

We assumed that the participants followed the instructions and tried their best to trace the trajectory. Therefore, the predicted/expected position of their hand should be on the trajectory. The visual prediction error was the average distance between the visual stimulus (i.e., the ball in HMD) and the trajectory. In the aligned condition, it equals the motion errors shown in Fig. 5B. In the offset condition, it equals half of the motion errors shown in Fig. 5B. A 2 × 2 (delay × visual modification) repeated measures ANOVA on visual prediction error revealed a significant main effect of delay (*F*(1, 14) = 25.546, *p* < 0.001, partial η^2^ = 0.646) and a significant main effect of visual modification (*F*(1, 14) = 116.399, *p* < 0.001, partial η^2^ = 0.893). The interaction between delay and visual modification was not significant (*F*(1, 14) = 2.509, *p* = 0.136, partial η^2^ = 0.152). The results confirmed that although the motion error was larger in the offset condition, the visual prediction error was indeed smaller than in the aligned condition, which was associated with higher agency ratings.

### Discussion

Experiment 1 examined the hypothesis that modification to visual feedback can improve the sense of agency during continuous body movements. In the experiment, participants rated their sense of agency while moving a visual target with their hand, which represented the position of their right hand. The offset condition reduced the discrepancy between the hand position and the trajectory by half. The results supported the hypothesis by showing improved agency ratings in the offset condition, even when the actual motion error was larger in the offset condition compared to the aligned condition. In addition, the interaction between delay and visual modification on the agency rating was nonsignificant. This was different from our prediction. We predicted that the effect of modification should be larger when motor control was more disturbed. This was probably because the motion errors in the no-delay condition was not minimal, and the visual modification also reduced perceived prediction errors and enhanced the sense of agency in the no-delay condition to a certain extent. Taken together, the results indicated the possibility of artificially improving the sense of agency during continuous body movements by slightly modifying visual feedback. Improvement in sense of agency may potentially lead to stronger motor intention and better recovery of voluntary motor control. However, in the real world, difficulty in motor control is unlikely to be caused by a delay in visual response, and is more often caused by physical disability. To further examine the hypothesis, in Experiment 2, a 1-kg weight was attached to the participants’ wrist to disturb motor control. Experiment 2 examined whether the hypothesis still holds when the motor impairment is physical rather than caused by delayed visual feedback.

## Experiment 2

### Participants

Fourteen participants took part in Experiment 2 (3 females, mean age = 21.8 years, *SD* = 2.4). All the participants had previously taken part in Experiment 1. Experiment 2 was conducted about 5 months after Experiment 1. The experiment was conducted with the approval of the ethics committee of the Faculty of Engineering at the University of Tokyo, and was performed in accordance with relevant guidelines and regulations. Written informed consent was obtained from all participants.

### Experimental task and design

**Figure 7:**
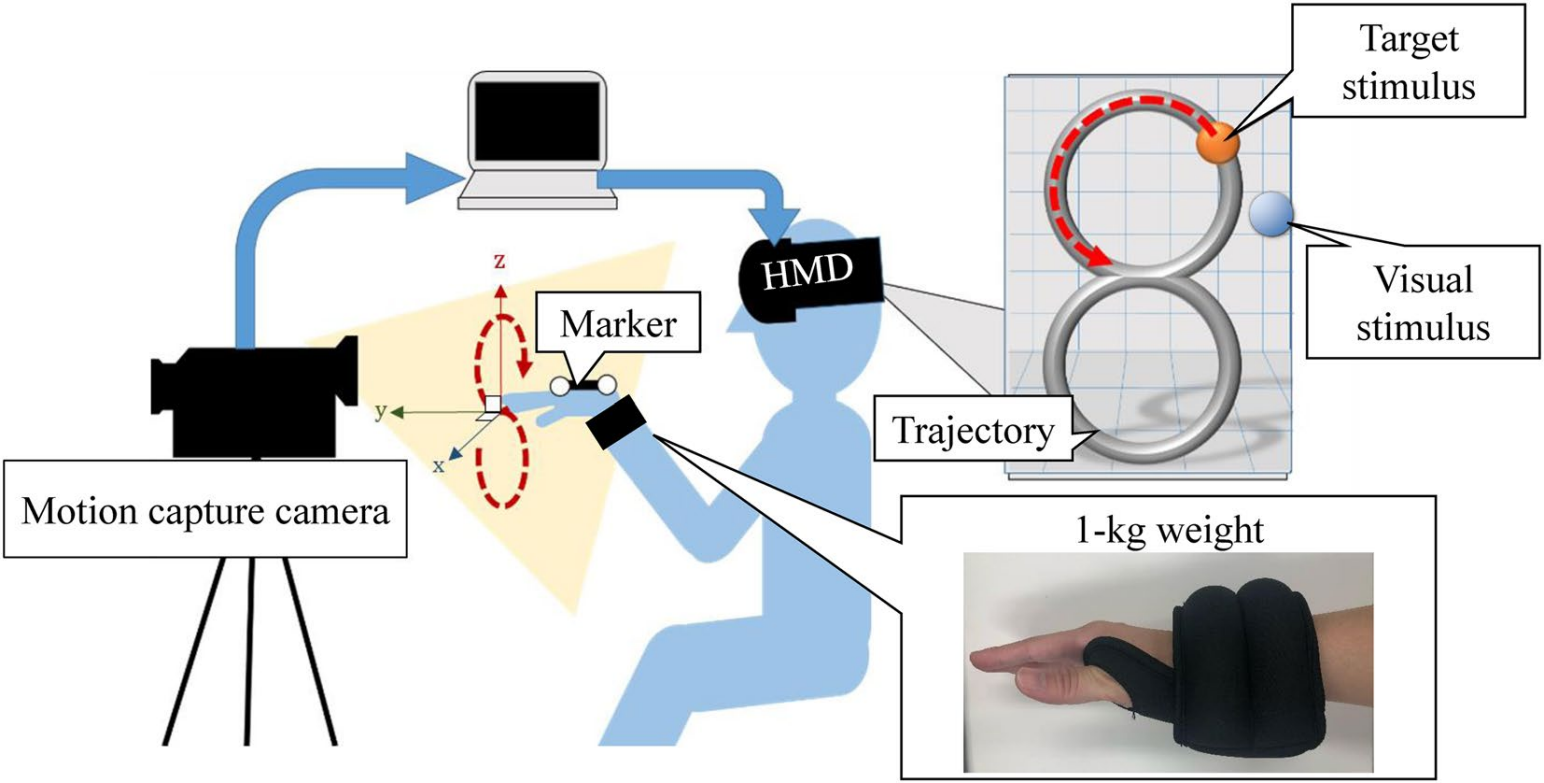
Configurations, stimuli, and the weight used in Experiment 2.

Figure 7 shows the system configuration and the weight used in Experiment 2. The task in Experiment 2 was similar to that in Experiment 1, except for the following points. First, there was no delay in the motion of the visual stimulus. Instead, a 1-kg weight (Nike, Inc.) was attached to the wrist of the participants’ right wrist to disturb motor control (Fig. 7). This weight was selected according to the results of our pilot experiments, confirming that it was sufficient to disturb participants’ motor control and participants would not be too fatigued to complete the experiment. Second, a vertical-eight-shaped trajectory was presented on a vertical surface in front of the participants, instead of the horizontal circular trajectory used in Experiment 1. The trajectory was parallel to the participants’ body surface. Moving on a vertical surface requires more effort while wearing the wrist weight, compared to moving on a horizontal surface. Moreover, vertical motor training is common in rehabilitation of motor impaired patients. The height of the trajectory was 40 cm, and the width was 20 cm. Third, a target stimulus was presented, which was an orange ball moving along the eight-shaped trajectory. The target stimulus moved at a constant speed along the trajectory. Participants were instructed to track the target stimulus along the trajectory by moving their hand. They were told to follow the target ball as closely as they can. The position of their own hand was represented by a blue ball (i.e., the visual stimulus). This was designed to better control the speed of movement. The target stimulus moved at a speed of 8 s per full circle.

There were three experimental conditions in Experiment 2. In the *baseline* condition, participants did not wear the wrist weight, and there was no modification to the visual feedback. The visual stimulus (i.e., the ball representing the participants’ hand position) corresponded to the actual position of the participants’ hand*.* In the *weighted & aligned* condition, the participants wore the 1-kg weight, and there was no visual modification. The visual stimulus represented the actual position of participants’ hands. Finally, in the *weighted & offset* condition, the participants wore the 1-kg weight, and the visual stimulus was presented at the median point between the actual hand position and the target stimulus.

### Procedure

The devices were the same as those used in Experiment 1, except for the additional weight. After receiving the instructions, the participants had two practice trials without the wrist weight. In the actual task, the participants first performed three trials of the baseline condition (without the wrist weight). Thereafter, the participants performed three trials of each of the weighted & aligned and weighted & offset conditions, in an alternating sequence. The order of the alternating sequence (i.e., starting from the weighted & aligned or weighted & offset conditions) was counter-balanced between participants. In summary, each participant performed nine trials in total, with each trial lasting 32 s (i.e., 4 full circles). After each trial, participants rated the question “How much did you feel that the ball in VR was controlled by your hand movements?” on a 7-point scale from 1 (not at all) to 7 (very much). The experiment took 60 min on average for each participant.

### Results

#### Agency rating

**Figure 8:**
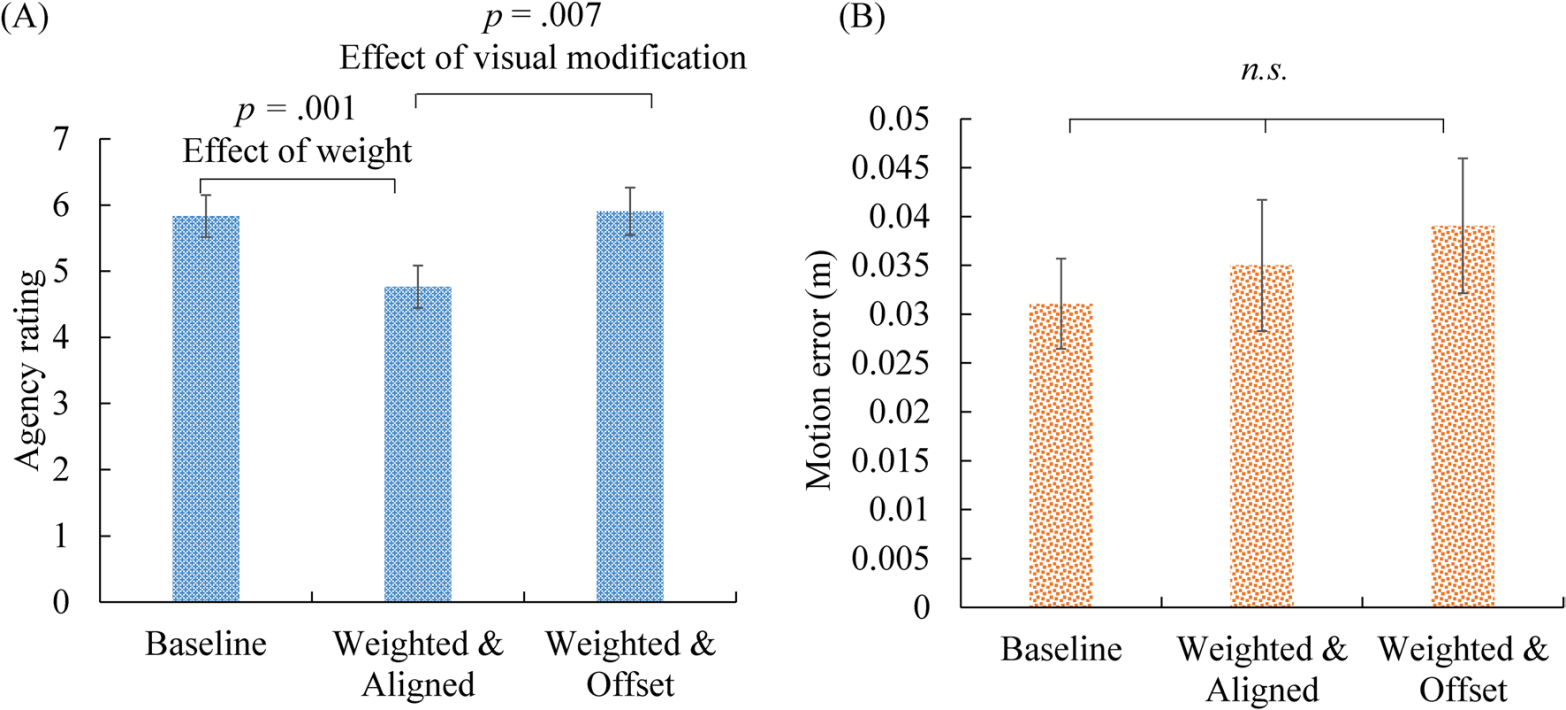
Agency rating (**A**) and motor error (**B**) in each condition of Experiment 2. Error bars represent standard errors.

Figure 8A shows the rating of sense of agency for each condition. We focused on two questions: whether the 1-kg weight impaired the sense of agency (baseline vs. weighted & aligned), and whether the visual modification improved the sense of agency (weighted & aligned vs. weighted & offset). We used the Kolmogorov–Smirnov test for each condition to check the normality of the data. The agency rating in the weighted & offset condition significantly differed from normal distribution (*d* = 0.278, *p* = 0.004). Therefore, related-samples Wilcoxon Signed Rank test was used for each comparison. The significance level was set to 0.025 (two comparisons were conducted: baseline vs. weighted & aligned, to examine the effect of weight; and weighted & aligned vs. weighted & offset, to examine the effect of visual modification) according to the Bonferroni correction. First, the comparison between the baseline and weighted & aligned conditions showed that the effect of weight on the sense of agency was significant (*Z* = 2.85, *p* = 0.004). The participants reported lower sense of agency over the visual stimulus due to the 1-kg wrist weight. Second, the comparison between the weighted & aligned and weighted & offset conditions showed that the effect of modification in the visual stimulus was also significant (*Z* = 2.36, *p* = 0.018). The visual modification significantly improved the sense of agency, even when the visual stimulus did not correspond to the exact position of the participants’ hand. In summary, the results from Experiment 2 again supported our hypothesis that modification of visual feedback that reduces visual prediction error can improve body agency.

#### Motion error

**Figure 9:**
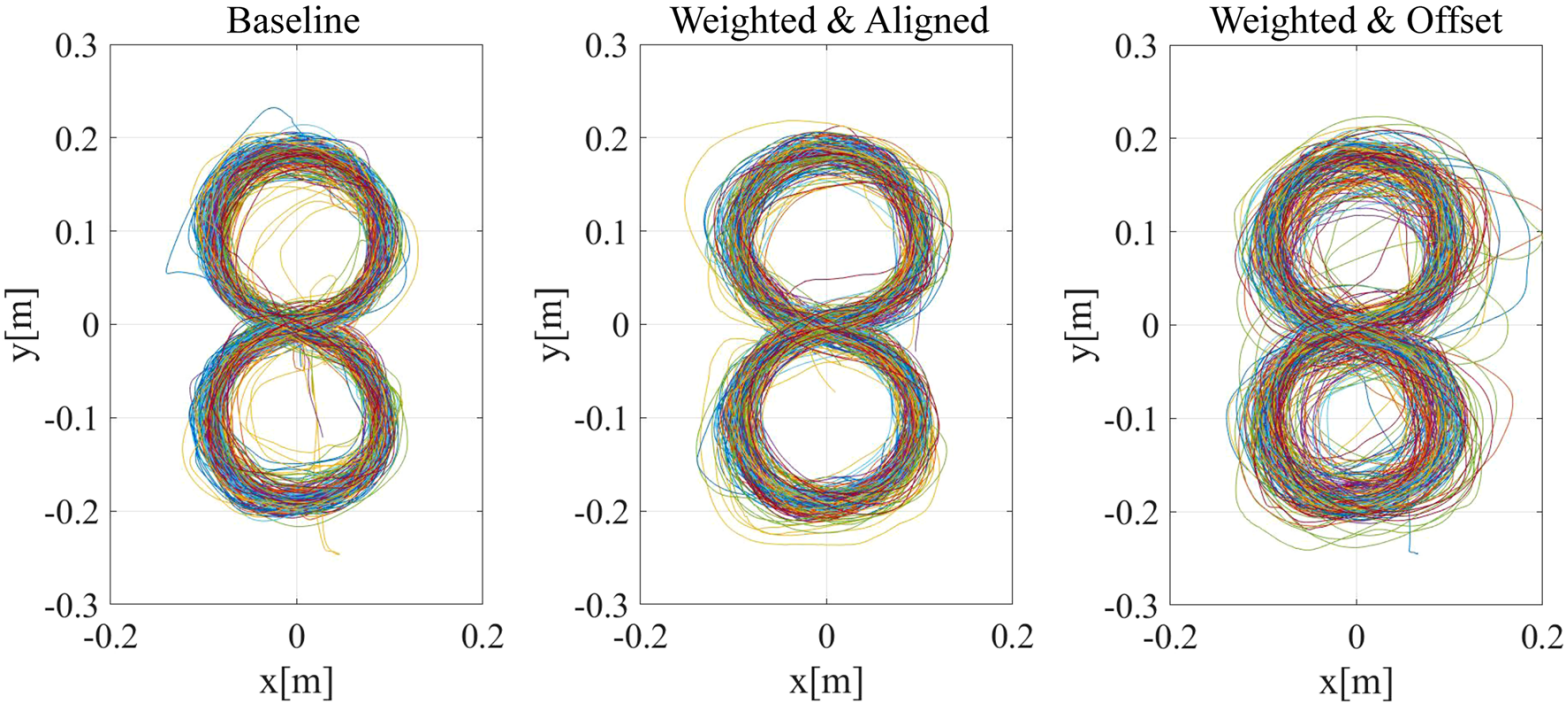
Plot of the participants’ hand movement trajectories in each condition in Experiment 2.

Figure 8B shows the distance between the actual position of the participant’s hand and the target stimulus, and Fig. 9 shows the actual trajectory of the hand movement in each condition. Since the motion trajectory used in this experiment was parallel to the participants’ body surface, the motion errors in the depth direction were difficult for the participant to perceive. Therefore, we excluded the error in the depth direction from the calculation, and only calculated the errors on the vertical surface of the trajectory. Kolmogorov–Smirnov test showed that the results in the weighted & aligned condition significantly differed from normal distribution (*d* = 0.343, *p* = 0.040). Therefore, related-samples Wilcoxon Signed Rank test was used for each comparison. The significance level was set to 0.025 according to the Bonferroni correction. Neither the difference between the baseline, and weighted & aligned condition, nor the difference between the weighted & aligned, and weighted & offset condition was significant (*Z* = 0.66, *p* = 0.510; *Z* = 1.79, *p* = 0.074, respectively).

#### Visual prediction error

The visual prediction error was calculated from the distance between the visual stimulus and the target stimulus. In the baseline and weighted & aligned conditions, visual prediction errors were equal to the motion errors. In the weighted & offset condition, visual prediction errors were equal to half of the motion errors. Related-samples Wilcoxon Signed Rank test was used for each comparison. The significance level was set to 0.025 according to the Bonferroni correction. The visual prediction errors were significantly smaller in the weighted & offset condition than in the weighted & aligned condition (*Z* = 3.30, *p* = 0.001). The difference between the baseline and the weighted & aligned conditions in visual prediction error was not significant (*Z* = 0.66, *p* = 0.510). The results showed that visual modification indeed reduced visual prediction errors. However, the results also showed that the decrease in agency rating in the weighted & aligned condition compared to the baseline was not purely due to the motion error or the visual prediction error, as these errors did not differ significantly between the two conditions. This might be due to more effort to exercise motor control in the condition in which people wore the weight compared to the condition in which they did not. Nevertheless, the effect of visual modification (non-modification vs. modification) on agency ratings showed that visually reducing prediction error indeed had a positive significant effect on the sense of agency.

### Discussion

In Experiment 2, a 1-kg weight was attached to the participants’ right wrist to disturb their motor control and the sense of agency. The results showed that although the precision of motion did not differ significantly among the three experimental conditions, the weight indeed weakened the participants’ sense of agency. More importantly, visual modification that artificially reduced the visual prediction errors significantly enhanced the sense of agency over the moving stimulus. People felt that they could better control the visual stimulus through their body movements, even though the visual stimulus did not correspond to their body movements. Taken together, the results from the two experiments indicate that externally reducing the error between one’s motor intention (i.e., the goal) and the visual feedback can significantly promote the sense of agency during continuous body movements, even when the greater discrepancy between the body movements and the visual feedback may actually be induced by such modification. These results highlight the possibility of external intervention of body agency for people whose motor control is impaired. The next section provides a detailed discussion of the findings and their implications.

## General discussion

The present study focused on the sense of agency, which is one of the critical components of voluntary motor control. We examined whether simple modification of visual feedback can enhance the sense of agency during continuous body movements when motor control is impaired. In the two experiments, the motor control of healthy participants was disturbed by either a delay in visual feedback (Experiment 1) or a 1-kg weight attached to their wrist (Experiment 2). Both methods significantly reduced the sense of agency during continuous body movements. In the offset conditions, the hand position was presented at the median point between the actual hand position and the desired hand position (depending on the goal of the motor task). Such modification in the visual feedback provided less accurate feedback of hand position, but significantly promoted the sense of agency. The results showed that the sense of agency during continuous body movements could be enhanced by simple modifications of visual feedback that reduce the perceived prediction errors.

In the present study, the use of VR blocked the direct visual input of one’s own body, and provided the possibility of replacing the visual feedback of one’s body movement with modified information. Many recent studies have used VR to study the embodiment and sense of agency of virtual bodies^29–33^. For example, studies using VR showed that the illusion of ownership of a virtual limb can be induced by synchronized visual-tactical stimuli, synchronous movement, and even brain-machine interface^30,34–38^. Furthermore, the illusion of ownership over a virtual limb can even influence the control of the real limb^20,39^. For instance, In Burin et al.’s study, participants watched a virtual hand drawing lines or ellipses^20^. When there was a mismatch between the intended and seen movements, participants’ movement was greatly “attracted” to the seen movements when they felt ownership over the virtual body^20^. By contrast, this study’s results showed that when the deviation did not conflict with their motor intention, people did not adjust their body movements to reduce such deviation. The motor errors were even bigger when the visual feedback deviated from the actual movements. Previous studies have shown that people tend to underestimate the deviation between their actions and visual feedback^40,41^, and the perceived position of the body can be influenced by modified visual body feedback^42–44^. Therefore, visual feedback probably dominated the perception of prediction errors. In addition, the high agency rating in the offset conditions revealed that the participants probably did not perceive much deviation between the position of their own hand and the visual feedback.

This study attempted to intervene in the sense of agency over continuous body movements using external visual modifications. Such intervention can be useful for patients with motor disabilities to improve their sense of agency during body movements, and can potentially benefit the updating of the internal model of motor control after sudden motor disabilities. When patients who have difficulties in body movement watch slightly more positive feedback of their body movements, which match their motor intention to a larger extent, it may enhance their sense of agency, and may benefit their planning and selection for the next move^9^. Smoother action selection may then benefit the sense of agency^45–48^, leading to a positive bootstrap between action selection and the sense of agency. Furthermore, previous research showed that motor disabilities in stroke patients are linked with body-specific attention decline, indicating dis-embodiment of the paretic limbs^49^. The intervening of the sense of agency among stroke patients may aid the re-embodiment of the limbs with motor disabilities and widen the peripersonal space^50^.

However, there are also issues in the measures of body agency in this study. Participants rated how much they felt that they were controlling the movement of the ball by moving their own hand. Such judgment may also be influenced by higher level factors such as intentions, beliefs, and inferences, besides lower levels of feeling of control^51^. Visually improved task-performance feedback can boost the rating of agency^52–54^. The positive task-performance feedback probably also contributed to the enhanced sense of agency. Furthermore, this study used a 600-ms delay in feedback and a 1-kg weight to disturb participants’ motor control. These motor disturbances and constrictions are probably much weaker than the motor disabilities that many patients have. The 1-kg weight did not significantly affect the precision of movement, although it reduced the sense of agency. This indicated that the effect of motor restriction in Experiment 2 was probably due to mental factors such as effort. It remains unknown if the proposed visual modification may improve the sense of agency in patients with more serious motor disabilities. Specifically, excessive visual modification might in turn reduce the sense of agency if the visual feedback differs from one’s own body movements too much. Finally, it is also unknown if the proposed visual modification does indeed have a positive effect on motor learning and motor intention, which is worthy of further examination in future research.

In conclusion, this study showed that the sense of agency during continuous body movements can be greatly affected when motor control is disturbed. More importantly, in such a condition, a simple modification of the visual feedback for body movements reduces perceived (visual) prediction errors and greatly enhances the sense of agency. These results uncover the possibility of enhancing body agency by external intervention to sensory feedback, indicating the prospect of a useful rehabilitation approach to improve the recovery of voluntary motor control in patients with acute motor disabilities.
